# A PICTORIAL PRESENTATION OF ESOPHAGEAL HIGH RESOLUTION MANOMETRY CURRENT PARAMETERS

**DOI:** 10.1590/0102-6720201700010019

**Published:** 2017

**Authors:** Fernanda M. LAFRAIA, Fernando A. M. HERBELLA, Julia R. KALLUF, Marco G. PATTI

**Affiliations:** 1 Department of Surgery, Escola Paulista de Medicina, Federal University of São Paulo, São Paulo, SP, Brazil; 2 Department of Surgery, University of North Carolina at Chapel Hill, Chapel Hill, USA

**Keywords:** Manometry, Classification, Esophagus, Esophagogastric junction, Esophageal motility disorders.

## Abstract

**Introduction:**

High resolution manometry is the current technology used to the study of esophageal motility and is replacing conventional manometry in important centers for esophageal motility with parameters used on esophageal motility, following the Chicago Classification. This classification unifies high resolution manometry interpretation and classifies esophageal disorders.

**Objective:**

This review shows, in a pictorial presentation, the new parameters established by the Chicago Classification, version 3.0, aimed to allow an easy comprehension and interpretation of high resolution manometry.

**Methods:**

Esophageal manometries performed by the authors were reviewed to select illustrative tracings representing Chicago Classification parameters.

**Results:**

The parameters are: Esophagogastric Morphology, that classifies this junction according to its physiology and anatomy; Integrated Relaxation Pressure, that measures the lower esophageal sphincter relaxation; Distal Contractile Integral, that evaluates the contraction vigor of each wave; and, Distal Latency, that measures the peristalsis velocity from the beginning of the swallow to the epiphrenic ampulla.

**Conclusion:**

Clinical applications of these new concepts is still under evaluation.

## RESUMO

A manometria de alta resolução é, atualmente, a tecnologia mais moderna para o estudo da motilidade esofágica e vem substituindo a manometria convencional nos grandes centros de pesquisa com parâmetros que seguem a Classificação de Chicago, que busca unificar as interpretações gráficas da manometria de alta resolução e, dessa maneira, categorizar os diversos distúrbios esofágicos.

**Objetivo:**

Mostrar, de forma pictórica, os novos parâmetros compilados na versão 3.0 da Classificação de Chicago, buscando facilitar a compreensão e interpretação da manometria de alta resolução.

**Métodos:**

Foram revistas as manometrias da casuística dos autores e selecionados os traçados representativos dos parâmetros da Classificação de Chicago.

**Resultados:**

Entre os parâmetros apresentados foram considerados a Morfologia da Transição Gastroesofágica, que classifica o segmento de acordo com sua fisiologia e anatomia; a Integral da Pressão de Relaxamento, que mede o relaxamento do esfíncter esofagiano inferior; a Integral Contrátil Distal, que avalia o vigor contrátil da onda peristáltica; e, a Latência Distal, que mede o tempo da peristalse, desde o início da deglutição até a ampola epifrênica.

**Conclusão:**

A aplicabilidade clínica desses novos conceitos ainda está sendo estudada.

## INTRODUCTION

High resolution manometry (HRM) is the current technology used to the study of esophageal motility. It is a tool that allows visualization of both sphincters and the esophageal body through a simultaneous and panoramic view from the pharynx to the stomach^11^.

HRM is replacing conventional manometry in important centers for esophageal motility due to its modern and technological features. It has been shown to be faster, more comfortable and devoid of certain limitations inherent to conventional manometry, as motion artifacts^4^. Therefore, it is essential that specialists and researchers are familiar with the parameters used in current studies. The parameters used on esophageal motility follow the Chicago Classification. This classification has been created by experts on esophageal motility and to unify HRM interpretation and classify esophageal disorders^2^. Chicago Classification is in its third version, published in 2015^5^. 

This review shows, in a pictorial presentation, the new parameters established by this classification aimed to allow an easy comprehension and interpretation of HRM studies. 

## METHODS

Esophageal manometries performed by the authors were reviewed to select illustrative tracings representing Chicago Classification parameters. 

## RESULTS

### Esophagogastric morphology

**FIGURE 1:**
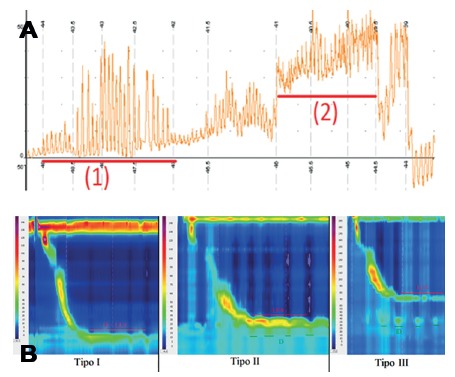
A) Hiatal hernia in conventional manometry: note the corresponding pressure zone to the diaphragm (1) and the corresponding pressure zone at the LES (2); B) morphology of the gastroesophageal junction in high resolution manometry: types are ranked according to the degree of dissociation of the diaphragm corresponding to the pressure (D) and lower esophageal sphincter (LES).

Conventional manometry defined hiatal hernia by the identification of two distal high pressure zones, corresponding to the diaphragm and the lower esophageal sphincter (LES)^11^ (Figure 1A). HRM has a higher accuracy that enables the distinction between diaphragmatic and LES pressures, even with great proximity or overlap of the two components. This allowed the description of three different types of esophagogastric morphology (Figure 1B)^12^. Type I is the complete overlap of diaphragmatic pressure and LES components with single peak on the spatial pressure variation plot. Type II is double-peaked pressure zone with the inter-peak nadir pressure greater than gastric pressure and a separation of 1-2 cm between peaks. Type IIIa is the separation greater than 2 cm between peaks, and nadir pressure less than or equal to gastric pressure. The pressure inversion point remains at the diaphragm level. Type IIIb is similar to type IIIa, but the pressure inversion point is at LES level.

**FIGURE 2:**
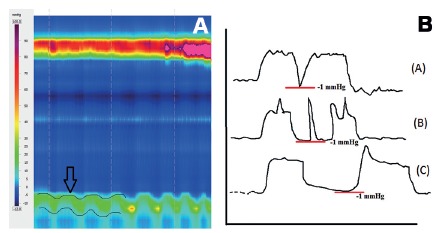
A) Lower esophageal sphincter respiratory oscillation: note that there is no dissociation of the components of the pressures corresponding to the diaphragm and lower esophageal sphincter, only respiratory motion (arrow); B) lower esophageal sphincter relaxation at conventional manometry measured by nadir pressure: pseudorelaxation due to factitious relaxation caused by sensor deeping into the stomach due to swallowing motion and not by actual relaxation of the sphincter; a short duration relaxation is noticed (A). In (B) diaphragmatic phasic contraction during relaxation may lead to misinterpretation of the relaxation duration, different from (C).

LES respiratory oscillation must not be interpreted as type II morphology (Figure 2A)^3^.

### Lower esophageal sphincter relaxation

**FIGURE 3:**
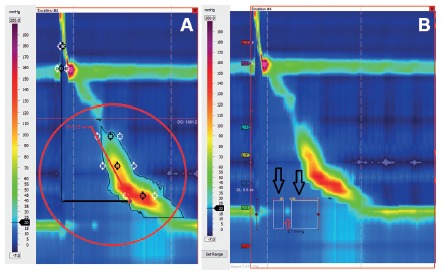
A) Measurement of lower esophageal sphincter relaxation by integrated relaxation pressure: note that the nadir pressure values are selected (black arrows), excluding the diaphragmatic pressure (red arrow), during 10 seconds after the beginning of the deglutition; B) measurement of contraction vigor by the distal contractile integral and note that the parameter is calculated considering the amplitude, duration and size of the contractile wave.

Conventional manometry measured LES relaxation at the nadir pressure^9^. This simple measurement may not distinguish a pseudorelaxation (Figure 2B)^6^. HRM permitted the creation of a new parameter, the Integrated Relaxation Pressure (IRP) that corresponds to the mean pressure of 4 s of greatest post-deglutive relaxation in a 10 s gap, triggered at the beginning of a swallow, which corresponds to the relaxation of upper esophageal sphincter (Figure 3A)^7^.

### Contraction vigor

Conventional manometry evaluated contraction amplitude at 3 cm, 8 cm, 13 cm and 18 cm of the LES superior border. Areas located in between sensors are not evaluated. HRM creates a panoramic view of the esophageal body allowing the evaluation and classification of the contraction vigor of each wave. For this purpose, the distal contractile integral (DCI) parameter was created. DCI value is calculated as the product of the mean amplitude of contraction in the distal esophagus (mmHg) times the duration of contraction (s) times the length of the distal esophageal segment (cm) exceeding 20 mmHg for the region spanning from the transition zone to the proximal aspect of the LES^17^ (Figure 3B). DCI classifies waves as ineffective, normal or hypercontractile^16^. 

### Peristalsis 

**FIGURE 4:**
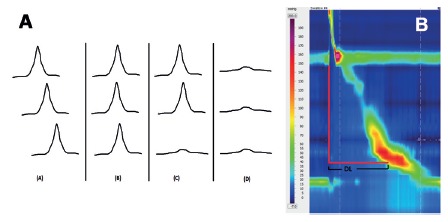
A) Types of waves at conventional manometry: (A) peristaltic, (B) simultaneous, (C) interrupted, (D) failed; B) measurement of peristalsis by distal latency (DL): note that the parameter is calculated by the time interval between the beginning of the swallow and the contractile deceleration point.

Conventional manometry classifies peristalsis based on the propagation and velocity of the waves (Figure 4A)^10^. HRM created the distal latency (DL) parameter that measures the peristalsis velocity from the beginning of the swallow to the epiphrenic ampulla. 

**FIGURE 5:**
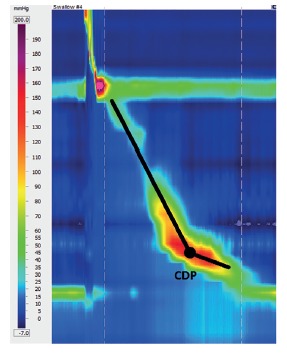
Identifying the contractile deceleration point (CDP): it corresponds to the transition from the esophageal body to the epiphrenic ampulla regarded as an inflection of the peristaltic axis in the topographic pressure graphic.

DL is the time interval between the beginning of the upper esophageal sphincter relaxation and the contractile deceleration point (CDP) ^14^ (Figure 4B). CDP is the manometric representation of the transition from the esophageal body to the epiphrenic ampulla regarded as an inflection of the peristaltic axis in the topographic pressure graphic, which corresponds to the place where a change in bolus propulsion speed occurs (Figure 5)^13^. CDP can be difficult to locate, therefore, Chicago Classification version 3.0 limited the localization of the CDP within 3 cm of the proximal margin of LES, in cases of atypical peristalsis^9^. 

## DISCUSSION

Chicago Classification is a new development that created new parameters and a new classification for esophageal motility disorders^2^. Following conventional manometry, some cases are unclassifiable in the standards defined by the Chicago Classification and a direct correlation between manometry and treatment is not always possible^1^. Version 3.0 is more clinical-oriented. A new version is anticipated for next year including the upper esophageal sphincter since this technology seems to be extremely advantageous for the study of this area^15^. 

## CONCLUSION

HRM brought new parameters for esophageal physiology study. Clinical applications of these new concepts are still under evaluation. 

## References

[1] Clarke JO, Pandolfino JE (2012). Esophageal motor disorders how to bridge the gap between advanced diagnostic tools and paucity of therapeutic modalities?. J Clin Gastroenterol.

[2] Herbella FA, Armijo PR, Patti MG (2016). A pictorial presentation of 3.0 Chicago Classification for esophageal motility disorders. Einstein (Sao Paulo)..

[3] Herbella FA, Vicentine FP, Del Grande JC (2010). High-resolution and conventional manometry in the assessment of the lower esophageal sphincter length. J Gastrointest Surg.

[4] Herbella FAM, Del Grande JC (2008). Novas técnicas ambulatoriais para avaliação da motilidade esofágica e sua aplicação no estudo do megaesôfago. Rev Col Bras Cir.

[5] Kahrilas PJ, Bredenoord AJ, Fox M, Gyawali CP, Roman S, Smout AJ, Pandolfino JE, International High Resolution Manometry Working Group (2015). The Chicago Classification of Esophageal Motility Disorders, v3 0. Neurogastroenterol Motil.

[6] Katz PO, Richter JE, Cowan R, Castell DO (1986). Apparent complete lower esophageal sphincter relaxation in achalasia. Gastroenterology.

[7] Lin Z, Kahrilas PJ, Roman S, Boris L, Carlson D, Pandolfino JE (2012). Refining the criterion for an abnormal Integrated Relaxation Pressure in esophageal pressure topography based on the pattern of esophageal contractility using a classification and regression tree model. Neurogastroenterol Motil.

[8] Lin Z, Pandolfino JE, Xiao Y, Carlson D, Bidari K, Escobar G, Kahrilas PJ (2012). Localizing the contractile deceleration point (CDP) in patients with abnormal esophageal pressure topography. Neurogastroenterol Motil.

[9] Martinez JC, Lima GR, Silva DH, Duarte AF, Novo NF, da Silva EC, Pinto PC, Maia AM (2015). Clinical, endoscopic and manometric features of the primary motor disorders of the esophagus. ABCD, arq. bras. cir. dig.

[10] Morais DJ, Lopes LR, Andreollo NA (2014). Dysphagia after antireflux fundoplication: endoscopic, radiological and manometric evaluation. ABCD, arq. bras. cir. dig.

[11] Pandolfino JE, Fox MR, Bredenoord AJ, Kahrilas PJ (2009). High-resolution manometry in clinical practice utilizing pressure topography to classify oesophageal motility abnormalities. Neurogastroenterol Motil.

[12] Pandolfino JE, Kim H, Ghosh SK, Clarke JO, Zhang Q, Kahrilas PJ (2007). High-resolution manometry of the EGJ an analysis of crural diaphragm function in GERD. Am J Gastroenterol.

[13] Pandolfino JE, Leslie E, Luger D, Mitchell B, Kwiatek MA, Kahrilas PJ (2010). The contractile deceleration point: an important physiologic landmark on oesophageal pressure topography. Neurogastroenterol Motil.

[14] Pandolfino JE, Roman S, Carlson D, Luger D, Bidari K, Boris L, Kwiatek MA, Kahrilas PJ (2011). Distal esophageal spasm in high-resolution esophageal pressure topography: defining clinical phenotypes. Gastroenterology.

[15] Rezende DT, Herbella FA, Silva LC, Panocchia-Neto S, Patti MG (2014). Upper esophageal sphincter resting pressure varies during esophageal manometry. Arq Bras Cir Dig.

[16] Roman S, Pandolfino JE, Chen J, Boris L, Luger D, Kahrilas PJ (2012). Phenotypes and clinical context of hypercontractility in high-resolution esophageal pressure topography (EPT). Am J Gastroenterol.

[17] Xiao Y, Kahrilas PJ, Kwasny MJ, Roman S, Lin Z, Nicodème F, Lu C, Pandolfino JE (2012). High-resolution manometry correlates of ineffective esophageal motility. Am J Gastroenterol.

